# The Acceptability of Virtual Characters as Social Skills Trainers: Usability Study

**DOI:** 10.2196/35358

**Published:** 2022-03-29

**Authors:** Hiroki Tanaka, Satoshi Nakamura

**Affiliations:** 1 Division of Information Science Nara Institute of Science and Technology Ikoma-shi Japan; 2 Data Science Center Nara Institute of Science and Technology Ikoma-shi Japan

**Keywords:** social skills training, virtual agent design, virtual assistant, virtual trainer, chatbot, acceptability, realism, virtual agent, simulation, social skill, social interaction, design, training, crowdsourcing

## Abstract

**Background:**

Social skills training by human trainers is a well-established method to provide appropriate social interaction skills and strengthen social self-efficacy. In our previous work, we attempted to automate social skills training by developing a virtual agent that taught social skills through interaction. Previous research has not investigated the visual design of virtual agents for social skills training. Thus, we investigated the effect of virtual agent visual design on automated social skills training.

**Objective:**

The 3 main purposes of this research were to investigate the effect of virtual agent appearance on automated social skills training, the relationship between acceptability and other measures (eg, likeability, realism, and familiarity), and the relationship between likeability and individual user characteristics (eg, gender, age, and autistic traits).

**Methods:**

We prepared images and videos of a virtual agent, and 1218 crowdsourced workers rated the virtual agents through a questionnaire. In designing personalized virtual agents, we investigated the acceptability, likeability, and other impressions of the virtual agents and their relationship to individual characteristics.

**Results:**

We found that there were differences between the virtual agents in all measures (*P*<.001). A female anime-type virtual agent was rated as the most likeable. We also confirmed that participants’ gender, age, and autistic traits were related to their ratings.

**Conclusions:**

We confirmed the effect of virtual agent design on automated social skills training. Our findings are important in designing the appearance of an agent for use in personalized automated social skills training.

## Introduction

Social skills training is a method widely applied to help people who lack social skills. It is used in medical hospitals, employment support facilities, workplaces, schools, and various other institutions [1]. Social skills training is generally conducted by a human trainer to promote appropriate social interaction skills and strengthen social self-efficacy [2]. The Bellack method (or step-by-step social skills training) is a well-structured and widely used evidence-based approach [1]. It is a cognitive behavioral approach to social skills training inspired by the 5 core principles of social learning theory: modeling, shaping, reinforcement, overlearning, and generalization [3]. The Bellack method defines the social skills training framework and its 4 basic skills: expressing positive feelings, listening to others, making requests, and expressing unpleasant feelings. These skills are beneficial for all people (not only those with autistic traits or schizophrenia) [1]. In particular, autism spectrum disorder (ASD) is a spectrum condition [4], meaning it has a broad range of characteristics, from mild to severe. Using computer agents in social skills training is motivated by the fact that even though some people with high-functioning autism experience difficulty during social communication, they also show good or even superior systemizing skills [5]. Systemizing is the drive to analyze or build systems and understand and predict behavior in terms of underlying rules and regularities. The use of systematic computer-based training for people who need to improve their social skills has the following benefits: (1) it uses a computerized environment that is predictable, consistent, and free from social demands; (2) users can work at their own pace and level of understanding; (3) training can be repeated until the goal is achieved; and (4) interest and motivation can be maintained through computerized rewards. It may also be easier for those who suffer from social difficulties to use computer agents than to directly interact with humans [6]. A past paper suggested that people with social difficulties such as ASD feel safer and more comfortable in virtual interactions than in interactions with actual people [7].

We and other research groups have been conducting studies to automate social skills training using virtual agents, and this work has led to the development of automatic social skills training [8-12] that by design resembles human-led social skills training [10]. The use of conversational agents in health care was reviewed by Tudor Car et al and Milne-Ives et al [13,14]. Among types of conversational agents, our system includes video modeling of human behavior, real-time behavior recognition, and feedback. We previously confirmed the effectiveness of this training in children and adults with ASD and in the general population. The automated social skills training agent plays 2 roles: as a trainer and as a listener. We confirmed that the system was more effective in training social skills than the traditional methods of reading books or watching videos of role models, and that talking to a 3D virtual agent made users feel more comfortable and less tense than talking to a human [15]. Automated social skills training targets various populations, from children to adult men and women, as well as those with ASD or schizophrenia [1]. However, visual designs of virtual agents, and what kind of design is more favored or more accepted, has not yet been investigated. A previous study showed that the quality of the therapeutic alliance (ie, the level of rapport and trust) is a reliable predictor of positive clinical outcomes independent of the approach to psychotherapy (including social skills training [1] and cognitive behavioral therapy [16]) or the specific outcome measure [17]. For automatic social skills training to be adopted and accepted by individuals, detailed investigation is necessary. In this study, we focus on comparing virtual agents with varying visual designs, rather than comparing humans and robots [18] for assistive technology [19], because we consider that the design of virtual agents is easier to create and modify.

The visual design of the virtual agent in social skills training has been previously investigated, although not exhaustively. For example, Hoque et al [12] paired male participants with a male coach and female participants with a female coach in order to minimize gender-based variability in behavior. By contrast, Tanaka et al [10,15] did not consider the agent’s gender (they used only a female design). Previous studies have used various virtual agent designs for different tasks and compared their appearance and behavior [20-22], realism [23], intensity in dialogue scenarios, and the appropriateness of body and eye proportions [24]. Past studies have also created a voice designed for the elderly [25] and have examined the impact of gender and race on users’ self-efficacy [26]. Troncone et al [27] discussed seniors’ psychological perspectives in terms of the model of acceptance and associated factors. Our study applies these findings and rating measures to investigate the design of our virtual agents, aiming to create a more favorable and acceptable design for automated social skills training. To the best of our knowledge, previous work has not investigated the visual design of virtual agents for automated social skills training, the relationship between acceptability and other measures, and the relationship between likeability and individual user characteristics.

This study set Japanese adults as our target users. We prepared a variety of new virtual agent designs for social skills training and evaluated them with multiple items on a questionnaire: their acceptability as a trainer; their acceptability as a listener; their realism, familiarity, trustworthiness, and eeriness; the likeability of their face, eyes, hair, perceived age, and voice; and their overall impression. These criteria were chosen with reference to the studies of Esposito et al [20,21,25] and Ring et al [24]. We followed their statistical analysis framework and investigated the appearance of 3D characters in the context of automated social skills training. First, we evaluated virtual agent visual design, particularly realism. Previous work has showed that serious tasks, such as medical diagnosis, require realistic agents; on the other hand, anime-like agents are more suited to social chitchat-like dialogue systems [24]. We hypothesized that anime-like characters would be preferable and more accepted for automated social skills training since such training requires friendly characteristics to maintain participant safety, and because agents play 2 roles: as trainers and as listeners. In addition, realism is affected by the “uncanny valley” phenomenon, with the most unrealistic character often being rated as the most acceptable [23]. We hypothesized that we would find that the uncanny valley also applies to automated social skills training agents. Second, to examine new factors that correlate to acceptability, we quantified the relationships between acceptability and other measures. We hypothesized that these questionnaire items would be highly correlated with each other [23]. Finally, we investigated the differences in preference for virtual agent design by considering individual users’ gender, age, and autistic traits in order to enable personalized automated social skills training. The three main research problems were (1) to investigate the visual appearance of virtual agents for automated social skills training; (2) to investigate the relationship between acceptability and other measures (eg, likeability, face, voice, realism, and familiarity); and (3) to investigate the relationship between acceptability and the individual characteristics of the user (ie, gender, age, and autistic traits).

This paper is an extension of conference proceedings [28] in which we reported on the visual design of characters. This paper adds an analysis of realism and includes a greater number of participants. We created new agents and videos and evaluated their realism. We also investigated whether people with high or low autistic traits rated the likeability of virtual agents differently depending on the realism of the agent. We also analyzed the correlation matrix between all questionnaire items in order to confirm correlations between acceptability and other measures. Finally, this paper discusses and summarizes findings from a series of experiments.

## Methods

### Visual Design of Virtual Agents

**Figure 1:**
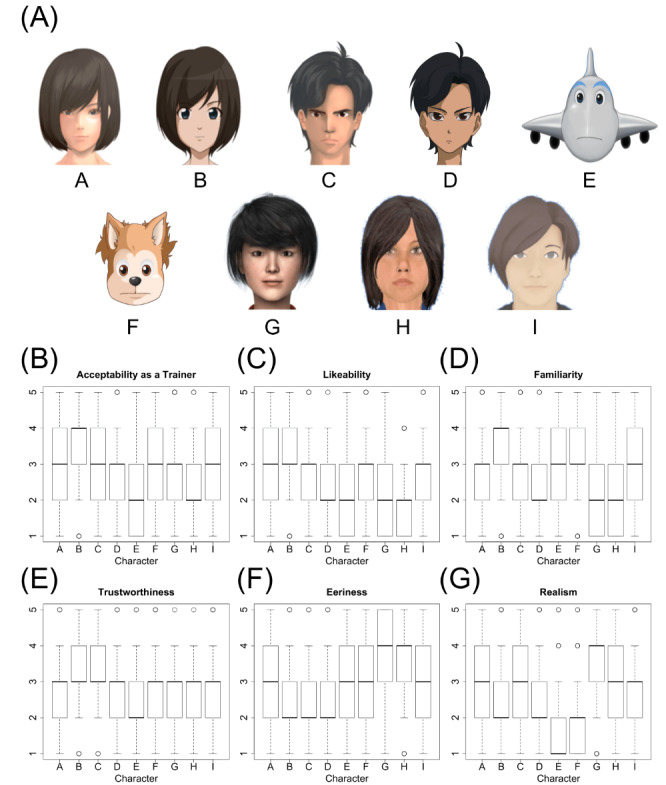
Images of the 9 virtual characters and representative measures collected from data set 1 and data set 2.

We first prepared an illustration of a virtual agent, as shown in Figure 1. The virtual agents were designed by a company specializing in Japanese animation.

All characters faced the front with no emotional expression. Characters A and B (female) and C and D (male) were designed with a consistent age, with only the degree of their realism and gender changing. Character E was an inanimate object created for use with children. Character F was a nonhuman animal (a dog), also for use with children. For character G, we created a realistic 3D model similar in appearance to characters A and B and took a screen capture from the front. Character H was the default agent provided by the Greta platform (developed by Pelachaud et al) [29], which is an embodied conversation agent that can be created with the Autodesk character generator (Autodesk Inc.) [30]. Character H was intended for use mainly with French- and English-speaking users. Character F was designed for Japanese female users. In the current study of automated social skills training, character I was selected as the virtual character [10]. The representation of characters H and I was created by taking a screen capture from the front.

The sentence “Hello, let’s practice communication together” was embedded in the image with both a male and a female voice. The utterance was 5 seconds in length and spoken by Google Text-to-Speech. Characters E and F were created with higher-pitched voices than those used for normal female speech synthesis, to mimic children’s voices.

**Figure 2:**
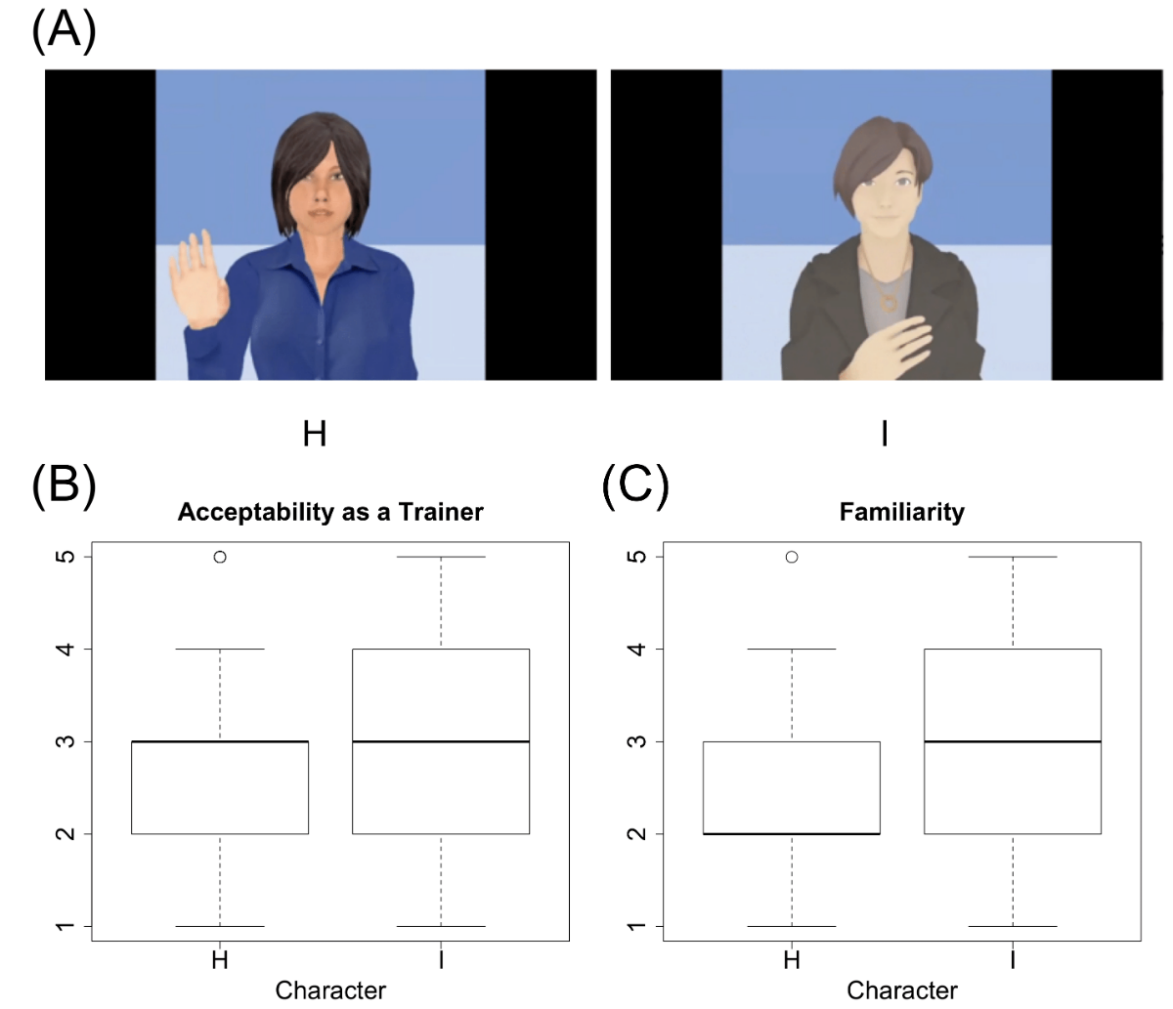
Screen captures of videos of two of the virtual characters (A) and representative measures collected from data set 3 (B, C).

Since 3D models were available for characters H and I, we were also able to create videos for them in Greta (Figure 2). Movements and gestures were added, such as the character raising its hands or putting its hands on its chest, synchronized to the speech content. These same behaviors and synchronization for characters H and I were also generated in Japanese, with an utterance length of 8 seconds. The speech synthesis used the voice of the character “Yuki” in CereProc (CereProc Ltd.).

**Figure 3:**
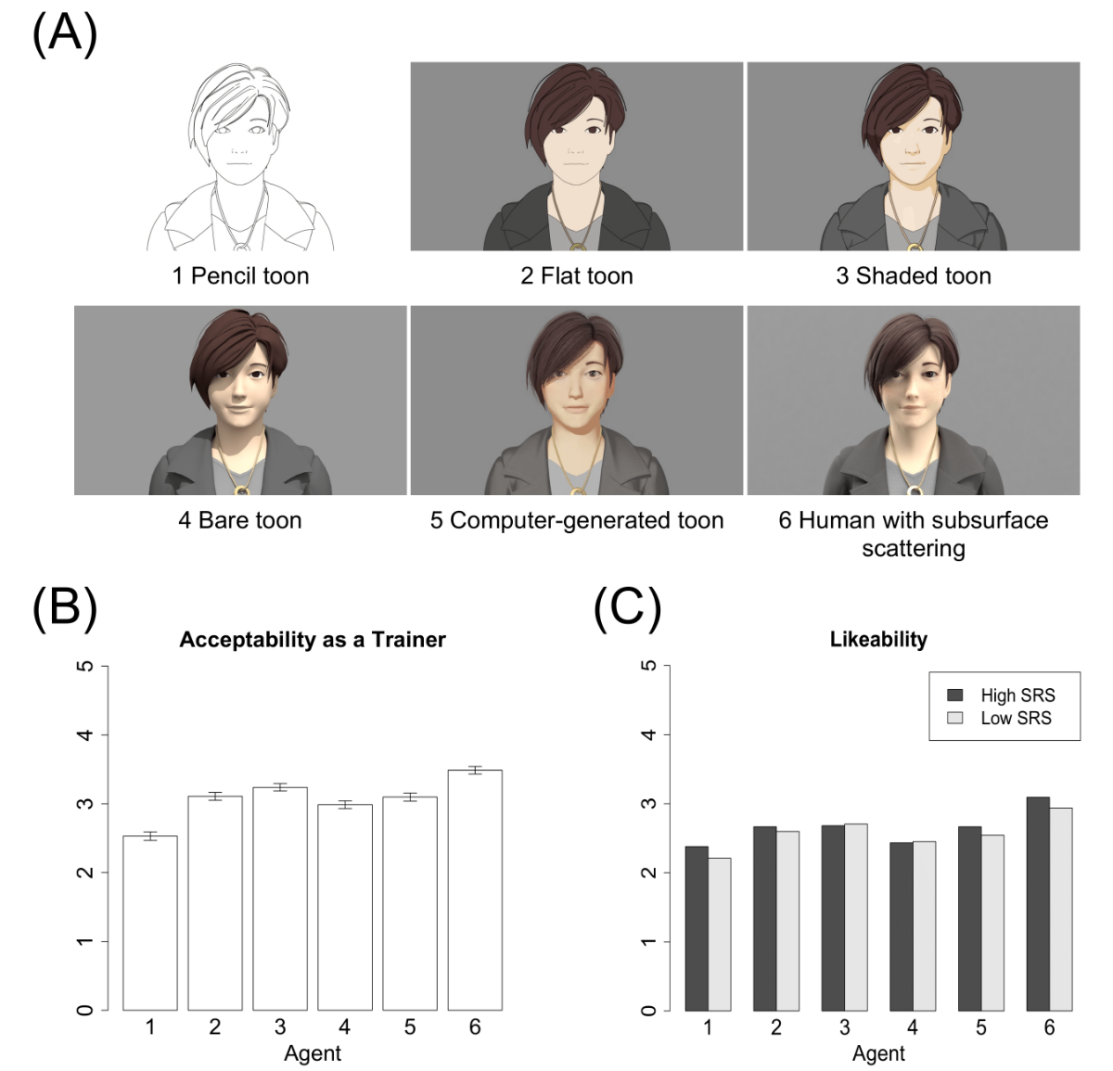
Screen captures of the 6 virtual agents (A); acceptability as a trainer in data set 4 (error bars represent SE) (B); and the evaluation of likeability by high and low SRS score groups (C).

We further analyzed the effect of realism by designing additional virtual agents, also with the aid of a design company specializing in Japanese animation. These agents were designed using the Maya tool (Autodesk Inc.). We prepared 6 levels of realism, following a previously reported method [23]. The degrees of realism were as follows: (1) pencil toon, (2) flat toon, (3) shaded toon, (4) bare toon, (5) computer-generated toon, and (6) human (with subsurface scattering), as shown in Figure 3. The same behavior was generated for these 6 agents, with Japanese speech synthesis and lip-synching using the same words as described above. All of these images and movies are available upon request to the first author.

### Participants

For data collection, we recruited participants from a crowdsourcing service (Crowdworks). The recruitment notice asked for participants 18 years of age or older with Japanese nationality. In order to divide the task among the participants, data were collected in 4 separate data sets with different participants. Data set 1 had 305 participants (with a male to female ratio of 148 to 157), data set 2 had 301 participants (with a male to female ratio of 131 to 170), data set 3 had 302 participants (with a male to female ratio of 145 to 157), and data set 4 had 305 participants (with a male to female ratio of 145 to 160). All data sets can be found in multimedia appendix. Data set 1 was used to investigate image acceptability, likeability, familiarity, likeability of certain elements (ie, eyes, face, hair, voice, and perceived age), autistic traits, and alexithymia. Data set 2 was used to investigate realism, trustworthiness, and eeriness of the agents. Data set 3 was used to investigate the videos with characters H and I. Data set 4 was used to investigate the realism of the movies, as well as autistic traits. For the validation to have a sufficient sample size, we collected a larger sample size for each data set compared to previous works, which have recruited around 40 to 70 participants from regional communities [20,21] or have used Amazon’s Mechanical Turk platform [24]. In this study, we also performed a grouped analysis using 45 years as the threshold for high and low age groups (high age: n=84; low age: n=21).

### Autistic Traits

In data set 1 and data set 4, we used the adult version of the Social Responsiveness Scale-2 (SRS) [31] to assess autistic traits. This measures how many autistic traits an individual shows and can be used across the general population, not only with people who are suspected of having ASD. In data set 1, we measured the Toronto Alexithymia Scale-20 (TAS) [32] to assess alexithymia. In both cases, we calculated the total score. We did not calculate subscales in this study. In data set 1, the 2 questionnaires had a Spearman correlation coefficient of 0.67 (*P*<.001), which indicates a high correlation between autistic traits and alexithymia. We are currently planning a future analysis that will use SRS as a measure of autistic traits. In this study, we used a cutoff value of 81 points [33] as the threshold for high and low SRS score (subjects with a high SRS score: n=113; low SRS score: n=192). We also measured SRS scores in data set 4 and also set a threshold for high and low SRS scores in that data set (high: n=129, low: n=177).

### Measures

Questionnaire items and scales were prepared with reference to studies by Esposito et al and Ring et al [20,21,24,25]. The questionnaire items measured the acceptability of the agent as a trainer and as a listener; its realism, familiarity, trustworthiness, and eeriness; the likeability of its face, eyes, hair, perceived age, and voice; and its overall impression. Each question was answered through a Google Form. In data set 1, each question item was answered after completing the SRS and TAS. In data set 3, in addition to the above, we added the likeability of the clothes the agent wore, because the video included the entire upper body of the virtual agent. We asked the participants to read a description of the concept of social skills training (in particular, the function of a virtual agent to train the user’s social communication skills and also listen to the user). We performed a preliminary test with a few adults to check whether the participants understood the social skills training, and we wrote instructions. Participants first looked at a set of all the images (Figure 1) to get an impression of all the virtual agents, and they then watched the individual virtual agents and answered each question. The questions were evaluated with a 5-point Likert scale (from 1, “I don’t think so at all,” to 5 “I think so very much”). Spearman ρ was calculated to determine the relationship between the questionnaire items.

R (R Foundation for Statistical Computing) was used for the analysis. Since normality could not be confirmed in the ratings of the questions by the Kolmogorov-Smirnov test, the Kruskal-Wallis test was used to examine the differences between the virtual agents. In the analysis for each group of gender, age, and SRS, we calculated the effect size (*r*). We report the top 3 combinations of *r* from all combinations of virtual agents and questionnaire items. Furthermore, we performed the Wilcoxon signed-rank test to compare pairs of factors.

### Ethical Considerations

This was an anonymous study in which the participants enrolled themselves by registering through Crowdworks and agreeing to participate in the study. Since participation was anonymized, the study was exempt from registration with our institutional review board.

## Results

In reporting the results, we did not report all measures, in order to focus on significant findings. The following is a summary of the experimental results.

First, the differences in ratings between the virtual characters. The Kruskal-Wallis test confirmed that there were significant differences between the virtual characters in all measures (*P*<.001). Regarding realism, the distribution was as expected in the original design: character A was more realistic than character B and character G was the most realistic. The most preferred virtual character among the participants was character B, averaging 3.29 (SD 1.0) (Figure 1). Character B was also highly evaluated in other questionnaire items. We also found that the male characters, C and D, and the nonhuman characters, E and F, had lower likeability than character B, and that character H had less likeability and less familiarity.

**Figure 4:**
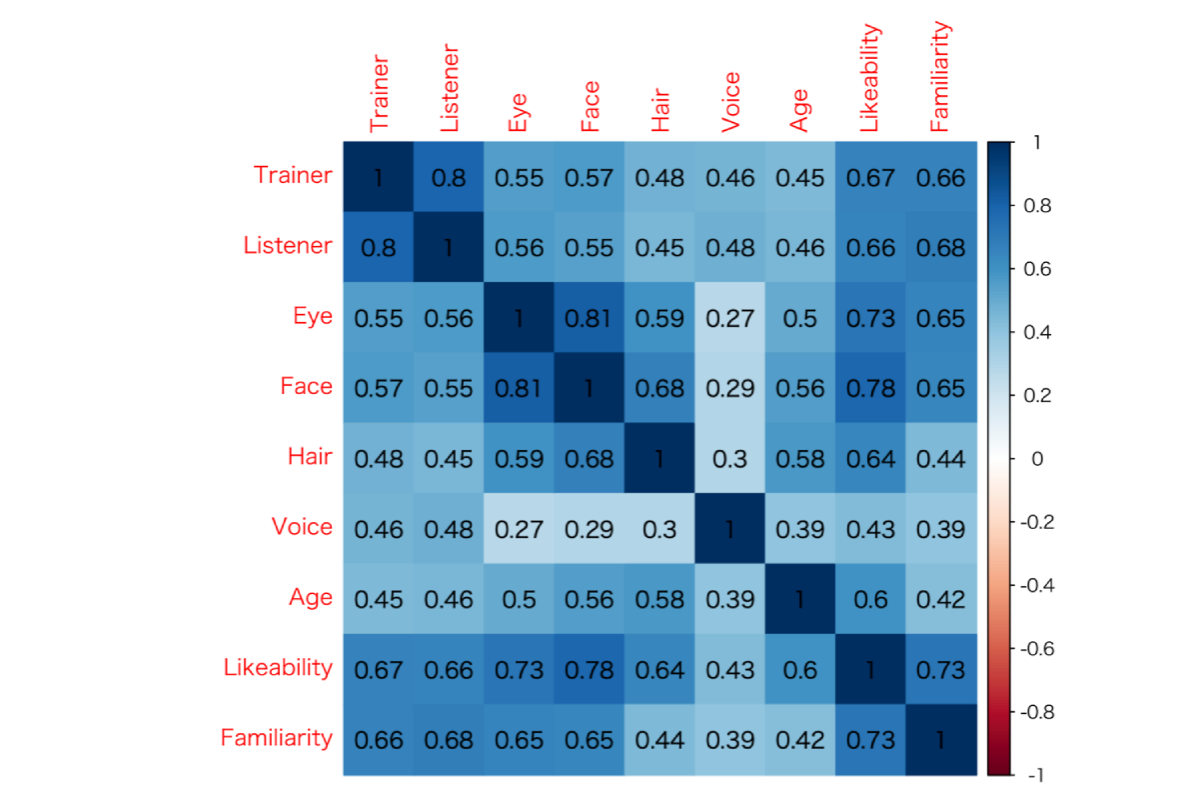
Correlation matrix of measures.

Next, the correlations between questionnaire items. Figure 4 shows the correlation matrix. There was a high correlation between face and preference (ρ=0.78, *P*<.001). There was also a high correlation between acceptance as a trainer and acceptance as a listener (ρ=0.80, *P*<.001). On the other hand, although a significant difference was confirmed regarding voice preference and other questionnaire items, the correlation coefficient was relatively low.

Table 1 lists the top 3 combinations of virtual agents and questionnaire items that had the highest effect size (*r*) for gender, age, and SRS score. All cases with a statistically significant difference are listed in Multimedia Appendix 1. Male subjects evaluated character G’s face, overall likeability, and acceptability as a trainer more highly than did female subjects. The higher age group evaluated character I’s eyes and face more highly than did the lower age group. The high SRS score group evaluated the likeability of character G’s eyes and hair more highly than did the low SRS score group.

Figure 2 shows a comparison of the videos of characters H and I, indicating that acceptability and familiarity were significantly greater for character I than H (all *P*<.001).

**Figure 5:**
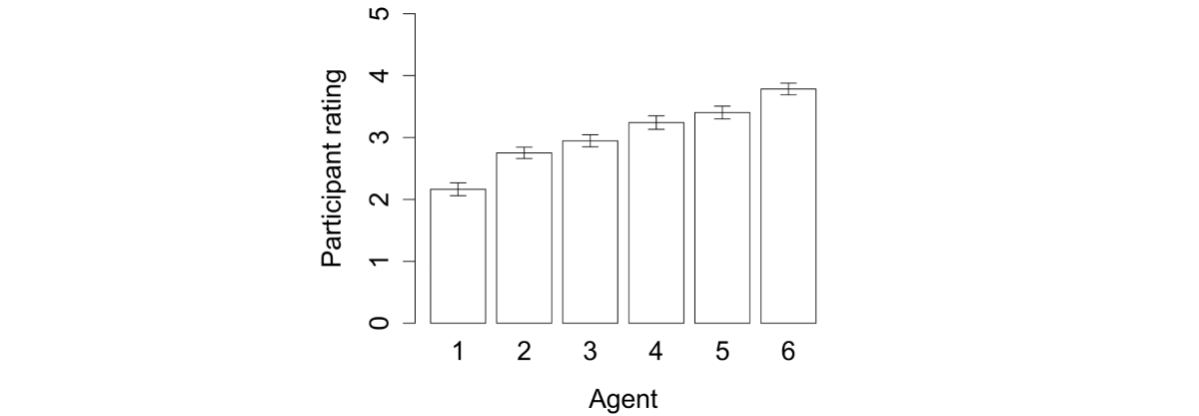
Realism measures collected from data set 4. Error bars represent SE.

Figure 5 shows the overall rating for realism for the agents shown in Figure 3. The Kruskal-Wallis test confirmed that the virtual agents differed significantly in realism (*P*<.001) and confirmed our design assumption that agents 1 through 6 would have increasingly greater realism. Figure 3 shows the acceptability as a trainer and likeability of the virtual agents. The Kruskal-Wallis test confirmed that the virtual agents differed significantly in all measures (*P*<.001). We found a small difference between the high and low SRS score groups in their evaluation of likeability (Figure 3 lower right), but the Wilcoxon rank-sum test showed no significant difference (for character 1, *P*=.13 and for character 6, *P*=.25) and a small effect size.

**Table 1:** Relationship between questionnaire items and gender, age, and SRS score.

User characteristic		Questionnaire item	*r* (*P* value)	Trend
**Gender**				
	Character G	Face	0.29 (<.001)	Male > female
	Character G	Likeability	0.25 (<.001)	Male > female
	Character G	Trainer	0.25 (<.001)	Male > female
**Age**				
	Character I	Eyes	0.21 (<.001)	High > low
	Character I	Face	0.19 (<.001)	High > low
	Character A	Listener	0.17 (.003)	High < low
**SRS score**				
	Character G	Eyes	0.19 (.001)	High > low
	Character G	Hair	0.18 (.002)	High > low
	Character G	Face	0.16 (.009)	High > low

## Discussion

The objective of this study was to examine virtual agent visual design for automated social skills training, the relationship between acceptability and other measures, and the relationship between likeability and individual user characteristics. We also investigated the acceptability and likeability of the virtual agents, as well as various other measures. We were able to confirm that the virtual agents had different ratings. First, we found that the realism of the virtual agent design could be controlled through the selection of characters A, B, or G. We found that character B, originally designed as an anime-like teenage female character, was the most likable (Figure 1). Since Japanese people are rather accustomed to watching anime-like videos, familiarity with such characters is high. The anime art form, having originated in Japan in the early 1900s, is a uniquely stylized form of 2D and 3D illustration [34]. Such a female anime-like character was also integrated and familiarized in our previous research on automated social skills training [8]. On the other hand, other virtual characters, such as the inanimate object (character E) or the animal (character F), as well as characters G and H, were less accepted and were not preferred.

We found significant correlations between questionnaire items (*P*<.001) and a high correlation between face and preference. This face factor influenced the development of the automated social skills training. There was also a high correlation between acceptance as a trainer and acceptance as a listener (Figure 4). In this case, we could not confirm the difference between the role as trainer and that as listener, because no continuous interactive dialogue was available. When the roles of virtual characters are more carefully chosen in the future, we assume that an investigation of this issue will also be necessary. Since the same voice was used for each virtual character, the correlation coefficient was relatively low. Therefore, we should explore the effect of voice using a variety of speech synthesizers in the future.

We also found very similar tendencies in video versions of the training agents. However, in terms of familiarity, we confirmed that the rating for the video version of character H was higher than its image version due to the addition of naturalistic movement. Regarding the videos shown in Figure 2, acceptability, and familiarity were significantly greater for character I than H. This shows that Japanese users preferred the anime-like character I over the original Greta character H.

We also found that realism, as shown in Figure 5, was associated with acceptability and likeability (Figure 3), a finding that is similar to that reported by McDonnell et al [23]. This may be related to the uncanny valley effect [35,36] and represent an intermediary between the responses to characters 3 (shaded toon) and 4 (bare toon). Although the most highly evaluated agent was character 6, the human with subsurface scattering, this sort of agent may need high-quality 3D modeling for its appearance and movement to be natural enough for use in automated social skills training. Thus, the second-ranked character, character 3 (shaded toon), may be the most promising for a realistic virtual agent for automated social skills training.

We found that the female virtual character, character G, was rated as more preferred by male participants. In addition, since we confirmed that character B was also significantly highly rated by male participants, it appears that the male participants rated female virtual characters as more preferable. Character B, originally designed as an anime-like teenage female character, was judged the most likable by all participants. We found that character I was preferred by older participants. Since character I was designed to appear relatively older (and was originally designed for participants in their 40s), it seems that the older group rated characters closer to their own age as more trustworthy. Therefore, when developing automated social skills training for older users, character I might be the most appropriate type of visual design. In this paper, one of our goals was to analyze the effect of autistic traits. We found that autistic traits were strongly associated with alexithymia (Spearman ρ=0.67). Thus, we focused only on SRS score to measure autistic traits. Our results showed that people with high autistic traits had a preference for realistic agents. We also confirmed that the group with high autistic traits gave a high rating to characters G and H in data set 1. This is a similar finding to previous work [18]. However, we did not find a difference in the case of data set 4.

Further investigation is needed to examine altered cognition in autism and its effects in order to conduct a comparison of virtual agents and real human agents. Although the target population of this study was adults 18 years or older, children with ASD may prefer nonhuman virtual agents, such as trains [5]. We must consider the effects of virtual agents in younger users. In future work, we hope to examine the effect of cultural differences, younger age, and virtual agent facial expressions on acceptability. These features could be used as variables of interest. In addition, this study did not confirm whether crowdsourced workers have sufficient knowledge of social skills training. Consequently, we need to investigate the effects of integrating design into an interactive social skills training dialogue system [8,9].

### Conclusions

In this study, we prepared various new virtual agent visual designs for social skills training and evaluated the designs based on multiple questionnaire items that assessed likeability, acceptability, realism, familiarity, and trustworthiness, among other factors, in a study sample of 1218 crowdsourced evaluators. We tested differences in preferences for virtual agent visual designs based on the gender, age, and autistic traits of the participants, in order to create personalized virtual agents. We found that our participants preferred, perhaps through familiarity, anime-like characters, likely because Japanese people are rather accustomed to watching anime-like videos. Our conclusion for implementing an optimal virtual agent for use with Japanese users is generally to design a female anime-type agent (especially a toon-shaded type), which has been shown to be favored and acceptable. We also found that preferences for virtual agent visual design differed according to user gender, age, and autistic traits. For example, we confirmed that users with high autistic traits showed a high preference for virtual agents with a realistic appearance.

## Appendix

Multimedia Appendix 1 All cases with a statistical difference.

Multimedia Appendix 2 Data set 1 to 4.
