# Supplementary material for: The Acceptability of Virtual Characters as Social Skills Trainers: Usability Study
Source: JMIR Hum Factors. 2022 Mar 29;9(1):e35358. doi: 10.2196/35358 (PMC9006137; doi:10.2196/35358)
Supplement: Multimedia Appendix 1 [file humanfactors_v9i1e35358_app1.docx]

Appendix. Questions related to gender, age, and SRS groups (all cases with statistical significance, ***: P < 0.001)

| Character | Question | r (P-value) | Trend |
| --- | --- | --- | --- |
| *Gender* |  |  |  |
| (g) | Face | 0.29*** | Male > Female |
| (g) | Likeablity | 0.25*** | Male > Female |
| (g) | Trainer | 0.25*** | Male > Female |
| (g) | Hair | 0.24*** | Male > Female |
| (g) | Listener | 0.24*** | Male > Female |
| (h) | Hair | 0.21*** | Male > Female |
| (g) | Eye | 0.21*** | Male > Female |
| (b) | Likeablity | 0.19*** | Male > Female |
| (a) | Eye | 0.19*** | Male > Female |
|  |  |  |  |
| *Age* |  |  |  |
| (i) | Eyes | 0.21*** | High > Low |
| (i) | Face | 0.19*** | High > Low |
|  |  |  |  |
| *SRS* |  |  |  |
|  | - | - | - |
